# Degradation mechanism of difructose dianhydride III in *Blautia* species

**DOI:** 10.1007/s00253-024-13346-5

**Published:** 2024-11-05

**Authors:** Ting Ye, Ayako Horigome, Hiroki Kaneko, Toshitaka Odamaki, Kanefumi Kitahara, Kiyotaka Fujita

**Affiliations:** 1 Faculty of Agriculture, Kagoshima University, 1-21-24 Korimoto, Kagoshima, Kagoshima, 890-0065 Japan; 2 Innovative Research Institute, Morinaga Milk Industry Co., Ltd, Research & Development Division5-1-83 Higashihara, Zama, Kanagawa 252-8583 Japan

**Keywords:** Difructose dianhydride III, Difructose dianhydride III hydrolase, *Blautia* spp*.*, Prebiotic, Gut microbe

## Abstract

**Abstract:**

Di-fructofuranose 1,2′:2,3′ dianhydride (DFA-III) is a cyclic fructo-disaccharide, which is produced by the condensation of two fructose molecules via the caramelization or enzymatic reaction of inulin fructotransferase. A strain of *Blautia producta* was known to utilize DFA-III as a carbohydrate source; however, the mechanisms remain unclear. In this study, we characterized the glycoside hydrolase (GH) family 91 DFA-III hydrolase (DFA-IIIase) from *B. parvula* NBRC 113351. Recombinant *Bp*DFA-IIIase catalyzed the reversible conversion of DFA-III to inulobiose, which is further degraded to fructose by the cooperative action of DFA-IIIase and GH32 β-d-fructofuranosidase. DFA-III was utilized in several *Blautia* species with a gene cluster for DFA-III degradation (e.g., *B. parvula* NBRC 113351, *B. hydrogenotrophica* JCM 14656, and *B. wexlerae* JCM 35486), but not by *B. wexlerae* JCM 31267, which does not possess the gene cluster. Furthermore, *B. hansenii* JCM 14655, which cannot metabolize fructose, could not utilize DFA-III; however, it could degrade DFA-III to fructose in the presence of DFA-III-degrading enzymes. Fecal fermentation tests showed that *Blautia* species are important gut microbe for degrading DFA-III.

**Key points:**

*• BpDFA-IIIase is the first characterized DFA-IIIase in intestinal non-pathogenic bacteria.*

*• DFA-IIIase is widely conserved in Blautia species.*

*• DFA-III is degraded to* *d* *-fructose through inulobiose by the cooperative action of DFA-IIIase and β-* *d* *-fructofuranosidase.*

**Supplementary Information:**

The online version contains supplementary material available at 10.1007/s00253-024-13346-5.

## Introduction

Difructose dianhydrides (DFAs) are cyclic disaccharides formed by the condensation of two fructose molecules and the removal of two water molecules. Tanaka et al. first reported α-d-fructofuranose-β-d-fructofuranose-1,2′:2,3′-dianhydride (DFA-III) formation by an extracellular enzyme from *Arthrobacter ureafaciens* (Tanaka et al. 1972). DFA-III can be produced from inulin by a DFA-III-forming type of inulin fructosyltransferase (IFTase) (EC 4.2.2.18), a member of the glycoside hydrolase family 91 (GH91) (Saito and Tomita 2000). In addition, enzymes for producing α-d-fructofuranose-β-d-fructofuranose-1,2′:2,1′-dianhydride (DFA-I) (Seki et al. 1989) and di-β-d-fructofuranose-2,6′:6,2′-dianhydride (DFA-IV) (Tanaka et al. 1983) have been characterized. However, only DFA-III is produced on an industrial scale. The physiological functions of DFA-III include enhancing mineral absorption (Mitamura and Hara 2005), reducing fecal secondary bile acid concentrations (Minamida et al. 2005a), and exerting positive effects on the immune system (Htun et al. 2018). In addition to being produced through enzymatic synthesis, DFAs can be formed in fructose-containing foods that are produced under high temperature and acidic conditions (Mellet and Fernández 2010).

DFA-III hydrolase (DFA-IIIase) (EC 4.2.1.178) was first characterized from *Arthrobacter* species (Tanaka et al. 1975). DFA-IIIase belongs to GH91 (Saito et al. 2003). DFA-IIIase is a right-handed β-helix, similar to IFTase, with a lid region covering the active center (Yu et al. 2018). In *Arthrobacter* species, DFA-III is hydrolyzed to d-fructose via inulobiose through a two-step hydrolysis pathway involving intracellular GH91 DFA-IIIase and GH32 β-d-fructofuranosidase (Sakurai et al. 1997; Tanaka et al. 1975). In addition to several DFA-IIIases from soil-isolated *Arthrobacter* species, a DFA-IIIase has been cloned and characterized from *Salmonella enterica* subsp*. enterica serovar* Mbandaka of the family Enterobacteriaceae (Yu et al. 2023) and *Duffyella gerundensis* A4 of the family Erwiniaceae (Yu et al. 2024).

*Blautia* is a genus of anaerobic bacteria belonging to the family Lachnospiraceae (*Clostridium* cluster XIVa) and is widely distributed in the mammalian gut. DFA-III increased the number of lecithinase-negative clostridia in the cecum of rats (Saito and Tomita 2000). DFA-III was also fermented in vitro by human feces (Kikuchi et al. 2004). *Blautia producta* (previously *Ruminococcus productus*) was isolated as a DFA-III-utilizing bacterium from the rat intestine (Minamida et al. 2006b). These findings suggest that *Blautia* species are major DFA-III-utilizing bacteria in human and rat intestines. However, the degradation mechanism of DFA-III in *Blautia* species remains unclear. Therefore, we performed an in vitro assimilation test using human fecal samples and *Blautia* strains isolated from human feces. In this study, we characterized GH91 DFA-IIIase as a key enzyme for DFA-III degradation in *Blautia* species.

## Materials and methods

### Materials

DFA-III was supplied from Nippon Beet Sugar Mfg., Co., Ltd. (Tokyo, Japan). Inulobiose was prepared from DFA-III by enzymatic treatment with DFA-IIIase from *Arthrobacter* sp. H65-7, as previously described (Sakurai et al. 1997). The chemical structures of substrates are shown in supplementary Fig. S1. Inulin from chicory roots was purchased from Bubble Star Co., Ltd. (Kanagawa, Japan).

### In vitro assimilation test of DFA-III by *Blautia* species

*B. hansenii* JCM 14655, *B. hydrogenotrophica* JCM 14656, *B. wexlerae* JCM 35486, and *B. wexlerae* JCM 31267 were obtained from Japan Collection of Microorganisms (JCM, Wako, Japan). *B. parvula* NBRC 113351 was obtained from the Biological Resource Center, National Institute of Technology and Evaluation (NBRC, Chiba, Japan). *B. parvula* NBRC 113351, *B. hansenii* JCM 14655, and *B. hydrogenotrophica* JCM 14656 are genome-analyzed strains that encode the GH91 DFA-IIIase gene. Draft genomic sequences of *B. wexlerae* JCM 31267 and JCM 35486 were determined and submitted to GenBank. DNA was extracted from the two strains and sequenced by bitBiome, Inc. (Tokyo, Japan; https://bitbiome.co.jp). Adaptors and low-quality reads were trimmed using bbduk v38.96. Trimmed reads were used for de novo assembly with SPAdes v3.15.2 (Prjibelski et al. 2020). Gene annotation was conducted using the web application of DFAST v1.6.0 (https://dfast.ddbj.nig.ac.jp) (Tanizawa et al. 2018). Taxonomic positions were determined using the digital DNA–DNA hybridization (formula d4) with the Type (Strain) Genome Server (TYGS) v358 (Meier-Kolthoff et al. 2022). The accession numbers are BTHI01000001–BTHI01000113 for JCM 35486 and BTHH01000001–BTHH01000071 for JCM 31267.

The cells were cultured in a 31-mL screw cap test tube sealed with a butyl rubber septum (Sanshin Industrial Co. Ltd., Kanagawa, Japan), and 10 mL of Gifu anaerobic medium (GAM) bouillon (#05422; Nissui Pharmaceutical, Tokyo, Japan) was used as the preculture medium. The loosely capped tubes were autoclaved at 121 °C for 15 min. After the autoclave temperature dropped to 97 °C, the tubes were immediately transferred into an anaerobic glove box (AS ONE Corporation, Osaka, Japan) filled with 100% nitrogen gas and placed until the temperature of the medium dropped below 37 °C. *Blautia* strains were inoculated into the medium. The test tubes were tightly capped and incubated at 37 °C. The in vitro assimilation test was performed using a GAM medium with or without carbohydrate. GAM semisolid without dextrose (#05460, Nissui Pharmaceutical) was dissolved in distilled water. The supernatant without agar was mixed to a final concentration of 0.25% DFA-III, 1-kestose, fructose, and DFA-I as the sole carbon source and autoclaved at 121 °C for 15 min. The precultured strains were inoculated into culture medium and cultured at 37 °C under anaerobic conditions. Each culture medium was evaluated by measuring. The absorbance of each culture medium was measured at 660 nm at 19.5, 25.5, 43.5, and 49.5 h.

### Expression and purification of recombinant BpDFA-IIIase and β-d-fructofuranosidase

The sequence of LC508_RS12690 (termed as *Bp*DFA-IIIase gene) was obtained from the genome of *B. parvula* NBRC 113351(accession number: NZ_CP084061.1). The codon-optimized sequence of *Bp*DFA-IIIase with a C-terminal His-tag was synthesized and cloned into a pET23a vector via GenScript (Nanjing, China) (Fig. S2). The synthesized pET23a–*Bp*DFA-IIIase plasmid was transformed into *E. coli* BL21 (DE3) cells. Cells were cultured using the Overnight Express Autoinduction System (Novagen) at 37 °C and then centrifuged. Cell pellets were resuspended in BugBuster protein extraction reagent (Novagen). Target proteins were purified on TALON metal affinity resin (Clontech Laboratories, Inc.) according to the manufacturer’s instructions. The purified fractions were desalted and concentrated using Vivaspin Turbo 15 with a 10-kDa cutoff (Sartorius Stedim, Gottingen, Germany). GH32 β-d-fructofuranosidase BBDE_1523 (GenBank ID: BAQ27517.1) from *Bifidobacterium dentium* was cloned and expressed following the same procedure.

### Enzymatic assays

For every type of substrate, enzyme activity was investigated by thin-layer chromatography (TLC) and high-performance anion-exchange chromatography with pulsed amperometric detection (HPAEC-PAD). In TLC analysis, the reaction products were spotted on Silica Gel 60 aluminum plates (Merck, Darmstadt, Germany) with a 5:3:2 (v/v/v) *n*-butanol/ethanol/water solvent mixture. The separated sugars were visualized by spraying an orcinol-sulfate reagent on the plates. For HPAEC-PAD, the reaction products were analyzed using a CarboPac PA1 column (Thermo Fisher Scientific). The column was eluted at a flow rate of 1.0 mL/min using the following gradient: 0–5 min, 100% eluent A (0.1 M NaOH); 5–30 min, 0–25% eluent B (0.5 M sodium acetate and 0.1 M NaOH); and 30–35 min, 100% eluent B.

*Bp*DFA-IIIase activity was assessed using DFA-III and inulobiose as the standard substrates in 40 µL of 50 mM acetate buffer (pH 6.0). Combination reaction of *Bp*DFA-IIIase and β-d-fructofuranosidase was also assessed using the same procedure. The reaction mixtures were incubated overnight at 37 °C and analyzed using TLC and HPAEC-PAD, as described. In addition, the reactivity of *Bp*DFA-IIIase was analyzed under the same reaction conditions using the following substrates: 1-kestose (GF_2_), nystose (GF_3_), 1^F^-β-fructofuranosylnystose (GF_4_), and inulin. The reaction mixtures were incubated overnight at 37 °C and analyzed using TLC.

### Activity of BpDFA-IIIase toward DFA-III and inulobiose

For equilibrium analysis, DFA-III (2.5 mM) or inulobiose (2.5 mM) was incubated in 50 mM acetate buffer (pH 6.0) with purified *Bp*DFA-IIIase (2.079 µg/mL) at 37 °C for different time points. The reaction was stopped by boiling for 3 min. The final equilibrium position was investigated individually. All assays were performed in triplicate.

### Effect of pH and temperature on BpDFA-IIIase activity

The optimal pH for *Bp*DFA-IIIase activity was determined using DFA-III as the substrate and 4.158 µg/mL of *Bp*DFA-IIIase as the enzyme between pH 3.5 and 8.0 in 50 mM sodium acetate buffer (pH 3.5–6.0) and 50 mM sodium phosphate buffer (pH 6.0–8.0). The optimal temperature of enzyme activity was determined using 50 mM sodium phosphate buffer (pH 6.0) at 20–55 °C. The reactions were stopped by adding 5% trichloroacetic acid, and the samples were analyzed using HPAEC-PAD. All assays were performed in triplicate.

### Determination of the kinetic parameters of BpDFA-IIIase

To determine the kinetic parameters of *Bp*DFA-IIIase, 4.158 µg/mL of *Bp*DFA-IIIase was incubated with 0.625–7.5 mM DFA-III or inulobiose in 50 mM of sodium phosphate buffer (pH 6.0) at 37 °C for 10 min. The reactions were stopped by adding 10 µL of 5% trichloroacetic acid, and the samples were analyzed using HPAEC-PAD. The values of the Michaelis–Menten constant (*K*_m_) and the maximum rate (*V*_max_) were estimated by a nonlinear regression method (Kitaoka 2023). All assays were performed in triplicate.

### Fecal fermentation and microbiota analysis

Fecal samples were cultured using a pH-controlled multichannel jar fermenter (Bio Jr.8; ABLE, Tokyo, Japan) in YCFA medium supplemented with 1.0% (w/v) DFA-III, as described previously (Murakami et al. 2021). Fecal samples collected from four healthy Japanese volunteers aged 28–45 years were cultured at 37 °C and collected at 0, 16, 24, and 48 h after fecal inoculation. DNA extraction, PCR amplification, and DNA sequencing for microbiota analysis were performed as described previously (Horigome et al. 2022).

### Gene distribution analysis

In July 2024, we retrieved 1376 genome sequences belonging to *Blautia* from the National Center for Biotechnology Information (NCBI). For analysis, we selected 718 sequences with > 95% completeness and < 5% contamination, as assessed by CheckM2 (Chklovski et al. 2023). We classified the filtered genome sequences into species using the GTDBtk v2.4.0 and GTDB Release 220 databases (Chaumeil et al. 2022). We predicted coding sequences from the genome sequences classified as *B. hansenii*, *B. hydrogenotrophica*, and *B. wexlerae* using Prodigal v2.6.3 (Hyatt et al. 2010). We conducted homology searches with GH91 sequences of each species using blastp v2.12 (Camacho et al. 2009). Because of the taxonomic synonymy between *B. parvula* and *B. celeris*, *B. celeris* genomes were classified as *B. parvula* genomes. Hits with similarity and coverage of ≥ 90% were counted as positive.

## Results

### In vitro assimilation of DFA-III by *Blautia* species

**Fig. 1:**
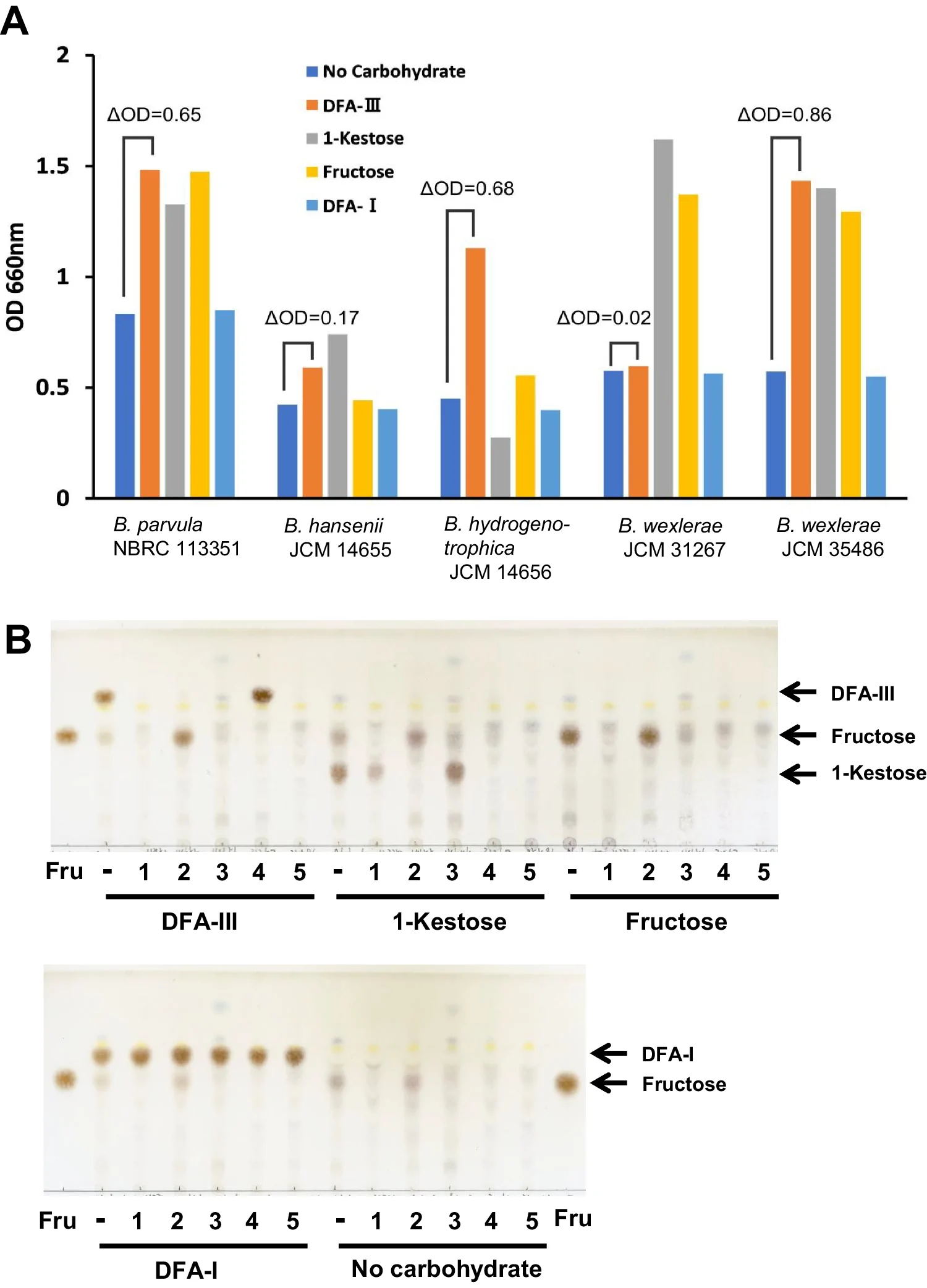
In vitro assimilation test of DFA-III. **A** The absorbance of *Blautia* strains after 43.5 h of culturing with 0.25% DFA-III, 1-kestose, fructose, or DFA-I as the sole carbohydrate source. The ΔOD indicates the difference in absorbance between DFA-III and carbohydrate-free media. **B** TLC analysis of residual carbohydrates in culture supernatants of *Blautia* strains after culturing for 49.5 h. Fructose was used as the standard. Lane 1, *Blautia parvula* NBRC 113351; lane 2, *B. hansenii* JCM 14655; lane 3, *B. hydrogenotrophica* JCM 14656; lane 4, *B. wexlerae* JCM 31267; and lane 5, *B. wexlerae* JCM 35486

For the assimilation tests, five *Blautia* strains were cultured in GAM medium containing 0.25% DFA-III, DFA-I, 1-kestose, and fructose as the sole carbohydrate source. The cells were cultured at 37 °C under anaerobic conditions. After 49.5 h of culturing, better growth of *B. parvula* NBRC 113351, *B. hydrogenotrophica* JCM 14656, and *B. wexlerae* JCM 35486 was noted (absorbance > 0.6) on DFA-III in comparison with no carbohydrate medium; however, *B. wexlerae* JCM 31267 exhibited no remarkable growth (absorbance < 0.2) on DFA-III medium (Fig. 1A). With the exception of strain *B. wexlerae* JCM 31267, no residual DFA-III was detected in the culture supernatant of DFA-III medium (Fig. 1B). In contrast, although *B. hansenii* JCM 14655 did not grow in DFA-III medium, it could degrade DFA-III to fructose. This strain has been known to exhibit a loss of fructose metabolism (Holdeman and Moore 1974). All strains, except *B. hydrogenotrophica* JCM 14656, could degrade 1-kestose, and DFA-I could not be degraded by any strain.

### DFA-III degrading gene clusters in *Blautia* species

**Fig. 2:**
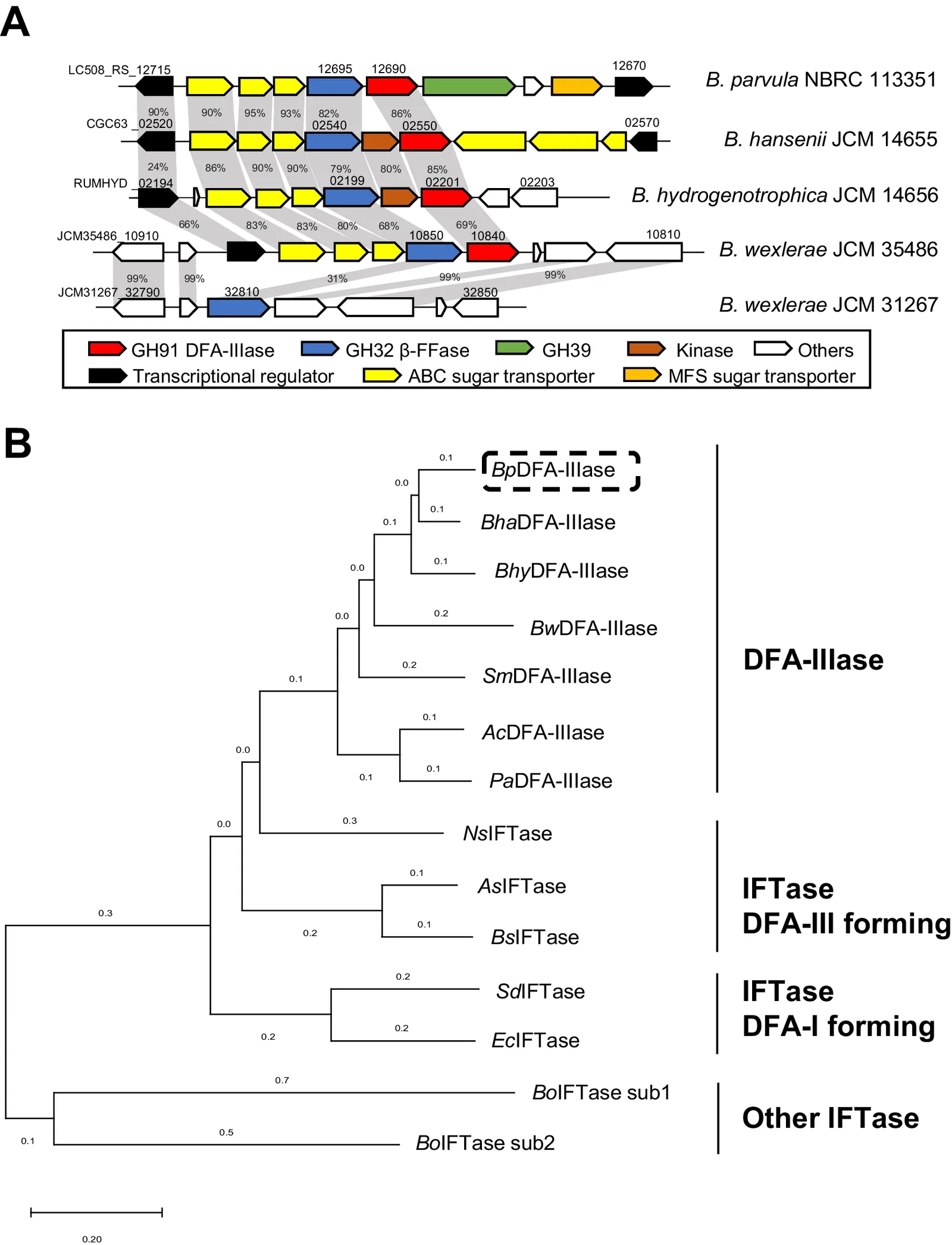
**A** Gene clusters involved in DFA-III degradation in *B. parvula* NBRC 113351, *B. hansenii* JCM 14655, *B. hydrogenotrophica* JCM 14656, and *B. wexlerae* JCM 35486. The gene clusters were compared with the corresponding cluster in *B. wexlerae* JCM 31267. Arrowheads with names indicate genes annotated in the Carbohydrate-Active EnZymes (CAZy) and Kyoto Encyclopedia of Genes and Genomes (KEGG) databases. Percentages in light gray bars indicate sequence identity between proteins from each strain. **B** Phylogenetic tree of GH91 enzymes. The phylogenetic tree was constructed using the neighbor-joining method and the aligned sequences. MUSCLE in MEGA7 software was used to construct the tree. The names of the characterized enzymes are shown next to the abbreviated names of the organisms as follows: *Bp*DFA-IIIase, *B. parvula* DFA-IIIase (GenBank ID: UBU24192.1); *Bha*DFA-IIIase, *B. hansenii* DFA-IIIase (ASM68477.1); *Bw*DFA-IIIase, *B. wexlerae* DFA-IIIase (GMN95340.1); *Bhy*DFA-IIIase, *B. hydrogenotrophica* DFA-IIIase (EEG48896.1); *Sm*DFA-IIIase, *Salmonella enterica* subsp. *enterica serovar* Mbandaka DFA-IIIase (HAB5395077.1); *Ac*DFA-IIIase, *Arthrobacter chlorophenolicus* DFA-IIIase (ACL40859.1); *Pa*DFA-IIIase, *Paenarthrobacter aurescens* DFA-IIIase (ADJ19283.1); *Ns*IFTase, *Nonomuraea* sp. IFTase DFA-III forming (BAN62836.1); *As*IFTase, *Arthrobacter* sp. IFTase DFA-III forming (BAA18967.1); *Bs*IFTase, *Bacillus* sp. IFTase DFA-III forming (AAZ66341.1); *Sd*IFTase, *Streptomyces davaonensis* IFTase DFA-I forming (CCK24566.1); *Ec*IFTase, *Enterocloster clostridioformis* IFTase DFA-I forming (WP_027644076.1); *Bo*IFTaseSub1, *Bacteroides ovatus* IFTase subunit 1 (ALJ46467.1); and *Bo*IFTaseSub2, *B*. *ovatus* IFTase subunit 2 (ALJ46468.1)

GH91 DFA-IIIase from *Arthrobacter* species is known to degrade DFA-III (Sakurai et al. 1997; Saito et al. 2003). Because *Blautia* species also contain conserved GH91 DFA-IIIase candidate genes, we compared gene clusters with the GH91 DFA-IIIase candidate gene across four *Blautia* strains, including *B. parvula* NBRC 113351, *B. hansenii* JCM 14655, *B. hydrogenotrophica* JCM 14656, and *B. wexlerae* JCM 35486, and one *B. wexlerae* JCM 31267 strain that does not contain the gene cluster (Fig. 2A). Because four *Blautia* species with the GH91 DFA-IIIase genes could degrade DFA-III (Fig. 1B), GH91 DFA-IIIase in *Blautia* must act as a key enzyme for DFA-III degradation. The GH91 DFA-IIIase candidate LC508_RS12690 (*Bp*DFA-IIIase) from *B. parvula* NBRC 113351 was found adjacent to a GH32 β-d-fructofuranosidase, an uncharacterized GH39 protein, and ABC transporters for DFA-III degradation. Genes from *B. hansenii* JCM 14655 (*Bha*DFA-IIIase) and *B. hydrogenotrophica* JCM 14656 (*Bhy*DFA-IIIase) were adjacent to a sugar kinase in addition to GH32 β-d-fructofuranosidase and ABC transporters. Interestingly, *B. wexlerae* JCM 35486 encoded a gene cluster that degrades DFA-III, including a DFA-IIIase (*Bw*DFA-IIIase) and GH32 β-d-fructofuranosidase and ABC transporters, whereas *B. wexlerae* JCM 31267 did not contain a gene cluster that degrades DFA-III, except for GH32 β-d-fructofuranosidase. The GH39 candidate gene may be not related to DFA-III degradation because it was not present only in *B. parvula* NBRC 113351. A phylogenetic tree was constructed using several sequences belonging to the GH91 family (Fig. 2B). *Bp*DFA-IIIase shared > 65% identity with characterized DFA-IIIases from *Arthrobacter chlorophenolicus* A6 (*Ac*DFA-IIIase), *Paenarthrobacter aurescens* SK 8.001 (*Pa*DFA-IIIase), and *S. enterica* subsp. *enterica serovar* Mbandaka (*Sm*DFA-IIIase). In addition, *Bp*DFA-IIIase shared > 71% identity with other DFA-IIIase candidates from *Blautia* species. *Bp*DFA-IIIase shared 43–49% identity with characterized IFTase (DFA-III forming), 41% identity with characterized IFTase (DFA-I forming), and 20–27% identity with an uncharacterized IFTase from *Bacteroides ovatus*. This indicates that *Blautia* enzymes belong to the DFA-IIIase subfamily along with other characterized DFA-IIIase from soil bacteria, and the DFA-IIIase subfamily is clearly distinguished from other GH91 family members.

### Characterization of recombinant BpDFA-IIIase

Recombinant *Bp*DFA-IIIase with a C-terminal His-tag was highly expressed as a soluble protein at 37 °C. Purified recombinant *Bp*DFA-IIIase migrated as a single band on SDS-PAGE with an apparent molecular mass of 50 kDa, which corresponded to its calculated molecular mass of 50,103 Da (Fig. S3).

**Fig. 3:**
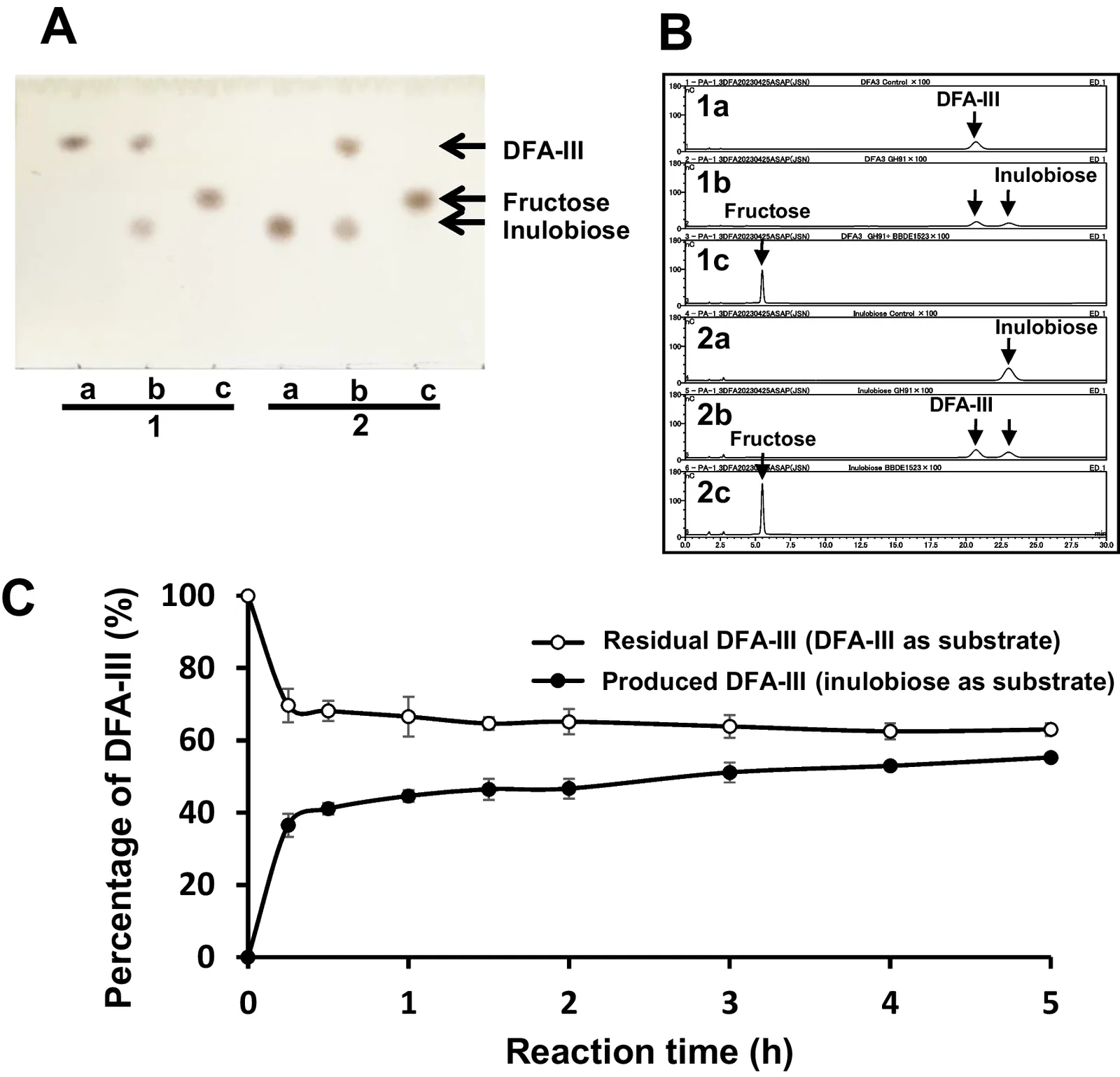
TLC (**A**) and HPAEC-PAD (**B**) analyses of the *Bp*DFA-IIIase reaction with DFA-III and inulobiose. The substrates (2.5 mM) were incubated overnight at 37 °C without (lane a) or with (lane b) *Bp*DFA-IIIase in 50 mM acetate buffer (pH 6.0). Lane c shows the reaction with *Bp*DFA-IIIase and β-d-fructofuranosidase under the same conditions. Lane 1, DFA-III; and lane 2, inulobiose. **C** Time course of the *Bp*DFA-IIIase reactions with DFA-III or inulobiose. DFA-III (2.5 mM) or inulobiose (2.5 mM) was incubated in 50 mM acetate buffer (pH 6.0) with purified *Bp*DFA-IIIase (2.079 µg/mL) at 37 °C

DFA-III and inulobiose were used as substrates for *Bp*DFA-IIIase (Fig. 3A, B). *Bp*DFA-IIIase catalyzed the conversion of DFA-III into inulobiose (lane 1b) and inulobiose into DFA-III (lane 2b) in an overnight reaction. In the presence of β-d-fructofuranosidase and *Bp*DFA-IIIase, DFA-III was completely hydrolyzed into d-fructose via inulobiose (lanes 1c and 2c). Thus, DFA-III is degraded into d-fructose via two steps of hydrolysis catalyzed by *Bp*DFA-IIIase and β-d-fructofuranosidase. Next, we examined the time-interval response of *Bp*DFA-IIIase with DFA-III or inulobiose. Although DFA-III and inulobiose were rapidly converted by *Bp*DFA-IIIase within 15 min, it took longer to reach equilibrium (Fig. 3C). After 24 h, the reaction reached an equilibrium ratio of DFA-III: inulobiose = 59.3:40.7. The kinetic parameters of *Bp*DFA-IIIase were determined by using inulobiose and DFA-III as substrates (Table 1). Apparent *K*_m_ values of *Bp*DFA-IIIase were lower for inulobiose than for DFA-III, and apparent *k*_cat_ values were higher for inulobiose than for DFA-III. Apparent *k*_cat_*/K*_m_ was 2.2-fold higher for inulobiose than for DFA-III. The higher catalytic efficiency to inulobiose reflects the equilibrium ratio of *Bp*DFA-IIIase.

**Table 1:** Kinetic parameter of *Bp*DFA-IIIase

	Apparent *K*_m_ (mM)	Apparent *k*_cat_ (s^–1^)	Apparent *k*_cat_/*K*_m_ (mM^–1^･s^–1^)
DFA-III	7.46 ± 0.10	1.39 ± 0.02	0.19
Inulobiose	4.93 ± 0.69	2.00 ± 0.81	0.41

**Fig. 4:**
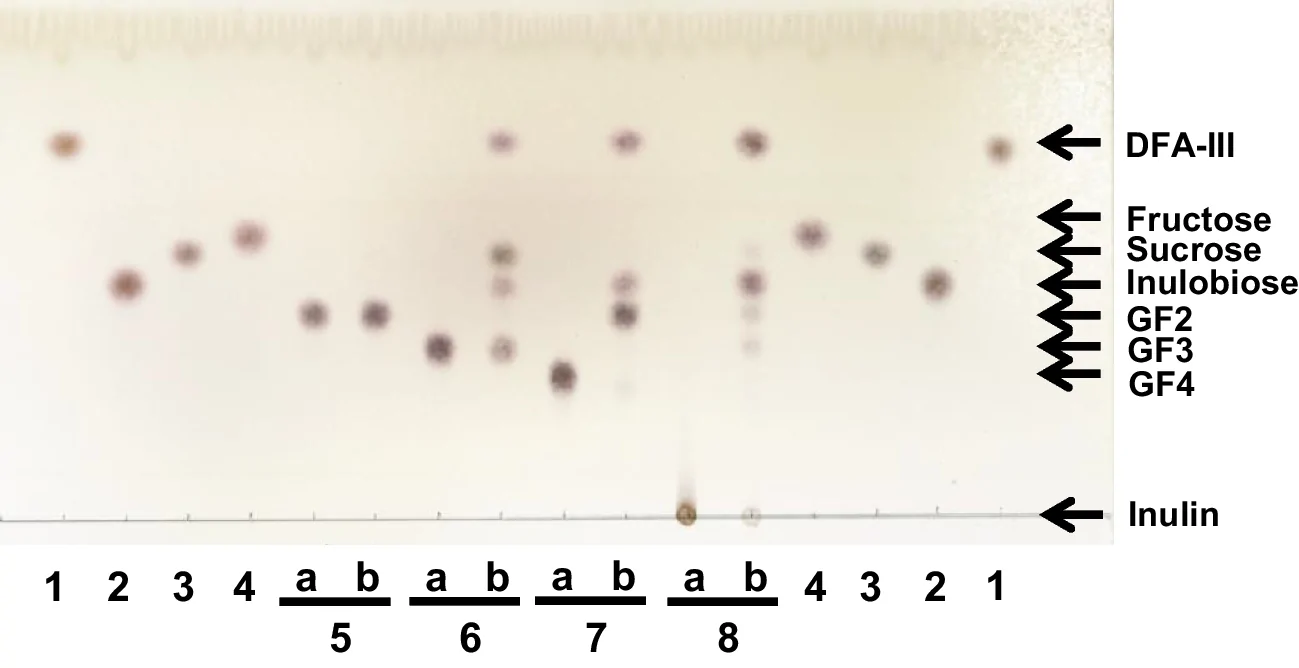
TLC analysis of substrate specificity without (lane a) or with (lane b) *Bp*DFA-IIIase. Lane 1, DFA-III; lane 2, inulobiose; lane 3, sucrose; lane 4, fructose; lane 5, 1-kestose (GF_2_); lane 6, nystose (GF_3_); lane 7, 1.^F^-β-fructofuranosylnystose (GF_4_); and lane 8, inulin. The substrates (2.5 mM) were incubated overnight at 37 °C in 50 mM acetate buffer (pH 6.0)

We tested the substrate specificity of *Bp*DFA-IIIase using GF_2_, GF_3_, GF_4_, and inulin as substrates (Fig. 4). *Bp*DFA-IIIase hydrolyzed GF_3_ into DFA-III, inulobiose, and sucrose (lane 6b); GF_4_ into DFA-III, inulobiose, and GF_2_ (lane 7b); and inulin to DFA-III and inulobiose (lane 8b). GF_2_ was not hydrolyzed (lane 5b). The specificity of *Bp*DFA-IIIase is similar to that of *Ac*DFA-IIIase (Yu et al. 2018). *Ac*DFA-IIIase can convert to IFTase by excluding the extra lid region. This lid allows *Ac*DFA-IIIase to hydrolyze DFA-III to inulobiose. *Bp*DFA-IIIase contains this conserved lid region (Fig. S2), which probably covers its active center, similar to that in *Ac*DFA-IIIase (Yu et al. 2018).

Furthermore, *Bp*DFA-IIIase did not hydrolyze DFA-I (data not shown). The optimal temperature and pH values for *Bp*DFA-IIIase were 35 °C and 6.0, respectively (Fig. S4). *Bp*DFA-IIIase activity was < 20% at pH 5.0 and 7.5 and decreased rapidly above 40 °C. *Bp*DFA-IIIase displayed stability over a narrow pH range and low thermal stability compared with *Sm*DFA-IIIase and *Ac*DFA-IIIase (Yu et al. 2023), which may be due to adaptation to the intestinal environment.

### Fecal fermentation abilities of DFA-III

**Fig. 5:**
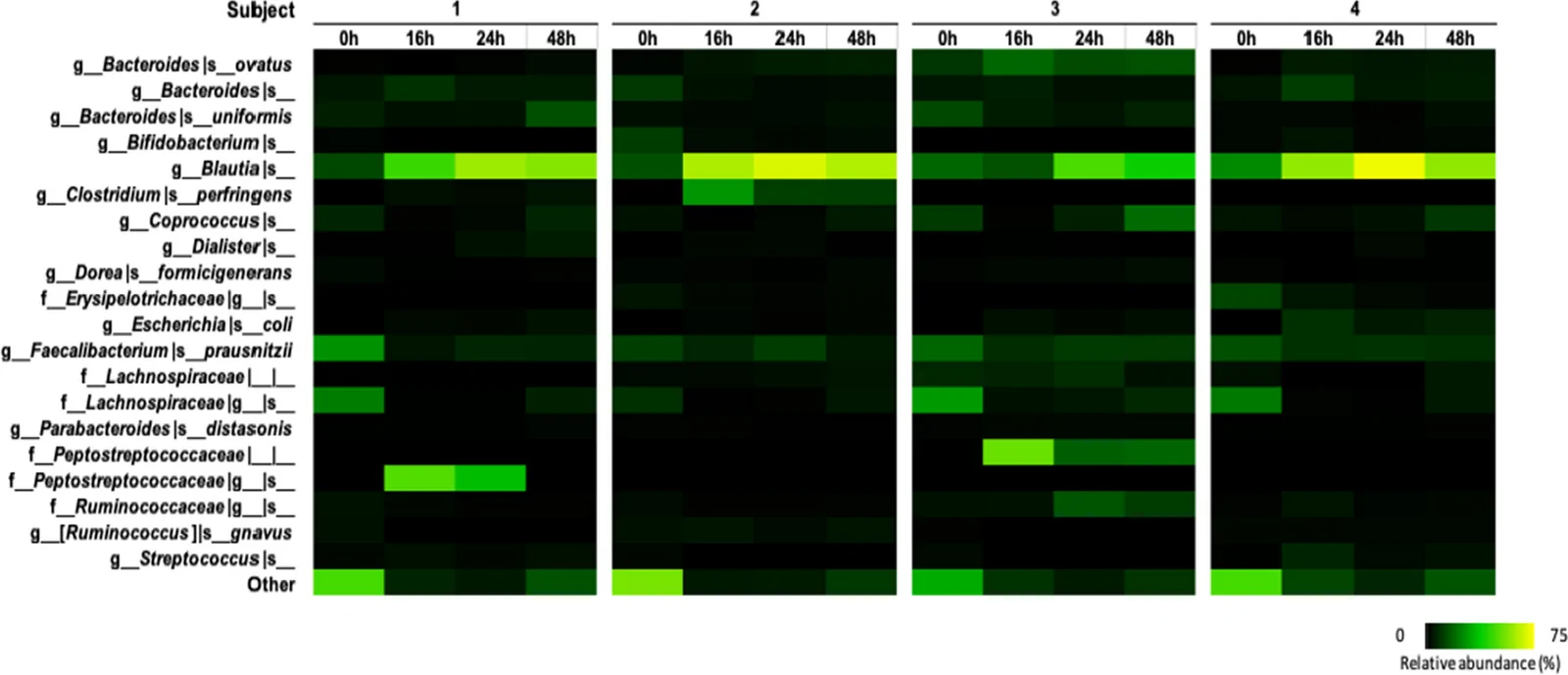
Composition of the fecal microbiota in each sample after fermentation in DFA-III. Relative abundances of the top 20 species-level taxa are shown; others are grouped into the “other” category

Fecal fermentation with DFA-III as a carbohydrate source was performed in the cultured fecal microbiota of four healthy adults using a pH-controlled single-batch fermenter (Fig. 5). The relative abundance of *Blautia* mainly increased in all fecal samples after 24 h. This indicates the potential of DFA-III as a prebiotic that can increase the abundance of *Blautia* in the human intestinal tract.

## Discussion

*B. producta* was the first strain to be characterized as a DFA-III hydrolyzing intestinal bacteria (Minamida et al. 2006b). In this study, we identified *B. parvula* NBRC 113351, *B. hydrogenotrophica* JCM 14656, and *B. wexlerae* JCM 35486 as DFA-III-utilizing bacteria. GH91 DFA-IIIase homologous genes were conserved in 66.7% of *B. parvula* strains (*n* = 21), 100% of *B. hydrogenotrophica* strains (*n* = 8), 50.0% of *B. hansenii* strains (*n* = 6), and 24.7% of *B. wexlerae* strains (*n* = 178) in the NCBI database. GH91 DFA-IIIase is widely conserved in *Blautia* species, with an overall conservation rate of 32.4%. However, the GH91 DFA-IIIase candidate is not conserved in *Bifidobacterium* and *Bacteroides* species, which are major carbohydrate-fermenting intestinal bacteria. According to a previous study, these bacteria could not ferment DFA-III in an in vitro fermentation test (Saito and Tomita 2000). Therefore, *Blautia* species are the major intestinal bacteria for DFA-III degradation present in the human gut. However, a member of *Klebsiella* and *Serratia* species of the family Enterobacteriaceae, as well as *S. enterica* subsp. *enterica serovar* Mbandaka (Yu et al. 2023), also encodes a DFA-IIIase homolog belonging to the GH91 DFA-IIIase subfamily.

*Bp*DFA-IIIase catalyzes the reversible reaction between DFA-III and inulobiose, reaching an equilibrium ratio of DFA-III:inulobiose = 59.3:40.7 at 37 °C and pH 6.0. The crude enzyme from *A. ureafaciens* ATCC 21124 showed an approximate equilibrium ratio of DFA-III:inulobiose = 60:40 at 30 °C and pH 6.0 (Neubauer et al. 2000). *Sm*DFA-IIIase and *Ac*DFA-IIIase showed an equilibrium ratio of DFA-III:inulobiose = 71:29 and 74:26 at pH 6.5 and 50 °C, respectively. Under the same pH and temperature conditions, the final equilibrium ratios of DFA-III and inulobiose were theoretically identical. The difference in the equilibrium ratio probably reflects the temperature.

In this study, we demonstrated that *Blautia* became a dominant bacterium in the presence of DFA-III using a pH-controlled single-batch fermenter with healthy human feces (Fig. 5). In an in vivo assay in rats, DFA-III supplementation increased the cecal abundance of *B. producta*, accompanied by a decrease in cecal pH and an increase in the content of short-chain fatty acids (SCFAs), especially acetic acid (Minamida et al. 2005b). Intestinal acidification in rats may inhibit the formation of secondary bile acids (Minamida et al. 2005a). This increase in acetic acid was confirmed in other studies showing DFA-III-feeding in rats (Afsana et al. 2003; Hira et al. 2017). Although a predominant increase in *Blautia* was not confirmed in the human DFA-III-feeding study, a tendency toward an increase in organic acids and a decrease in secondary bile acids was observed (Minamida et al. 2006a). We have previously shown that the abundance of *Blautia* varies across human fecal cultures in responders and non-responders using 2′-fucosyllactose (Horigome et al. 2022), which indicates that DFA-III is a potential prebiotic that increases the abundance of *Blautia* in the human gut. However, it is influenced by the diversity of intestinal bacteria and the genetic characteristics of *Blautia*.

The potential of *Blautia* as a functional microorganism with probiotic properties has been widely recognized. *Blautia* plays important roles in metabolic diseases, inflammatory diseases, and biotransformation (Liu et al. 2021). *B. producta* ameliorated type 2 diabetes and was identified as an antihyperlipidemic probiotic (Tong et al. 2018; Wu et al. 2022; Xu et al. 2023; Yang et al. 2022). *B. wexlerae* ameliorated obesity and type 2 diabetes (Hosomi et al. 2022). *B. hansenii* was significantly and negatively associated with visceral fat accumulation (Ozato et al. 2022). *B. hydrogenotrophica* was reported improvement in bowel habit in irritable bowel syndrome (Quigley et al. 2022).

**Fig. 6:**
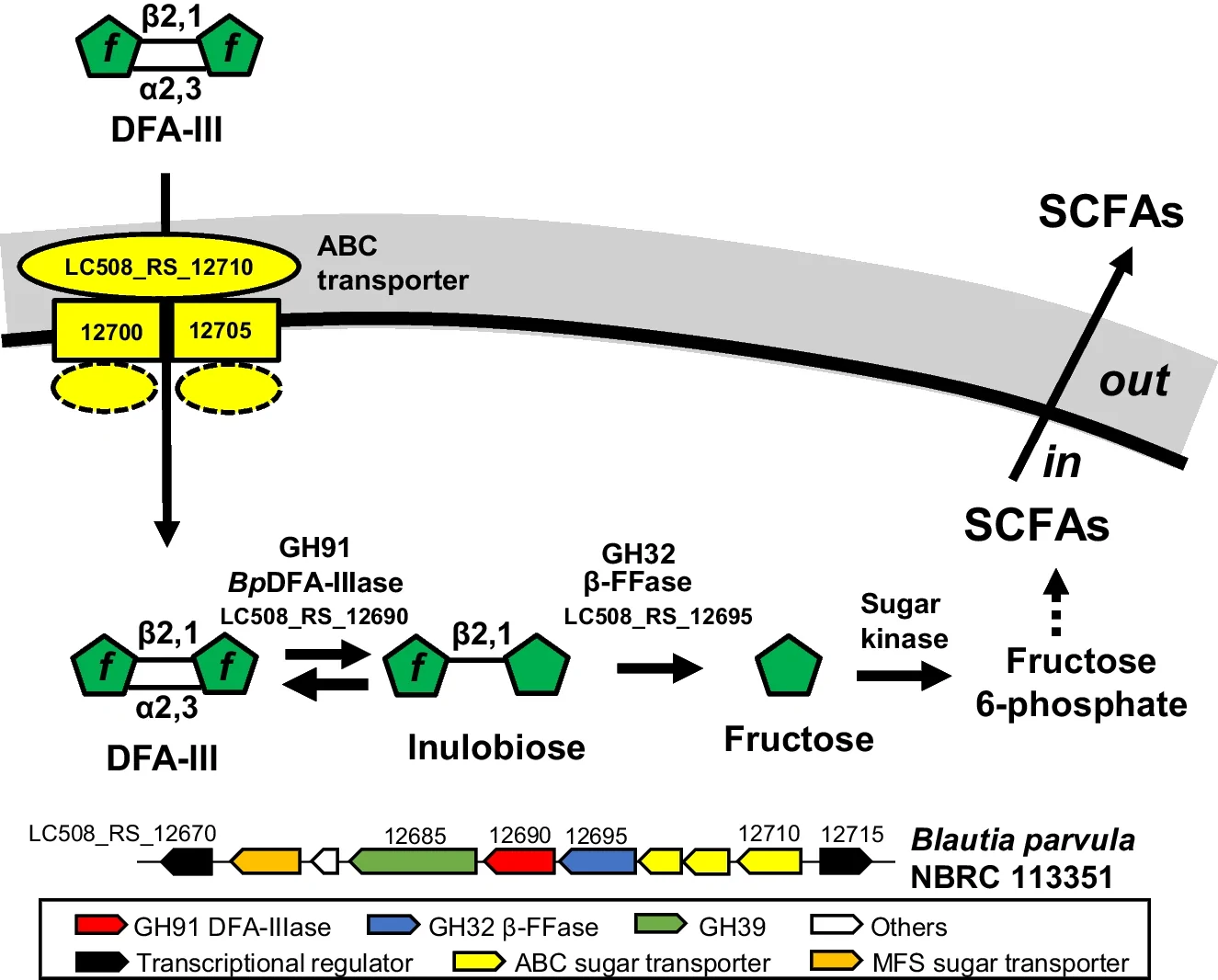
Schematic model of DFA-III degradation pathway in *Blautia parvula* NBRC 113351

Intestinal bacteria are dependent on extracellular carbohydrate and perform intracellular catabolism of carbohydrate hydrolysates to obtain energy for growth, with SCFAs as the main end products. Based on a previous study (Yu et al. 2018), we hypothesized that the degradation of DFA-III in *B. parvula* NBRC 113351 (Fig. 6) occurs as follows: (1) DFA-III is internalized via ABC transporters on the cell surface and reversibly converted to inulobiose by GH91 *Bp*DFA-IIIase. (2) Inulobiose is hydrolyzed to d-fructose by GH32 β-d-fructofuranosidase. (3) D-fructose is metabolized to SCFAs via glycolysis.

In conclusion, this study identified a key gene cluster containing GH91 DFA-IIIase for hydrolyzing DFA-III in *Blautia* species. Identification of a DFA-III-degrading pathway in *Blautia* species forms a basis for developing this species as next-generation probiotics. We have previously characterized a GH172 DFA-I hydrolase in bifidobacteria (Kashima et al. 2021). Caramelized sugars contain multiple DFAs and diheterolevulosans. Intestinal bacteria may perform fermentation cooperatively and competitively. Understanding the mechanisms of caramelized sugar hydrolysis by intestinal bacteria can help develop probiotic and prebiotic strategies.

## Supplementary Information

Below is the link to the electronic supplementary material.

Supplementary file1 (PDF 172 KB)

## Data Availability

The datasets generated during and/or analyzed during the current study are available from the corresponding author on reasonable request.
