# Supplementary material for: Degradation mechanism of difructose dianhydride III in Blautia species
Source: Appl Microbiol Biotechnol. 2024 Nov 5;108(1):502. doi: 10.1007/s00253-024-13346-5 (PMC11538131; doi:10.1007/s00253-024-13346-5)
Supplement: Supplementary file 1 — Supplementary file1 (PDF 172 KB) [file 253_2024_13346_MOESM1_ESM.pdf]

Journal: Applied Microbiology and Biotechnology

Degradation mechanism of difructose dianhydride III in *Blautia* species

Running Title: Characterization of *Blautia* DFA-IIIase

Authors: Ting Ye[1], Ayako Horigome[2], Hiroki Kaneko[2], Toshitaka Odamaki[2], Kanefumi Kitahara[1], Kiyotaka Fujita[1]*

* **Address correspondence to:** Kiyotaka Fujita, k4022897@kadai.jp

Affiliations:

1. Faculty of Agriculture, Kagoshima University, 1-21-24 Korimoto, Kagoshima, Kagoshima 890-0065, Japan

2. Innovative Research Institute, Research & Development Division, Morinaga Milk Industry Co., Ltd., 5-1-83 Higashihara, Zama, Kanagawa 252-8583, Japan

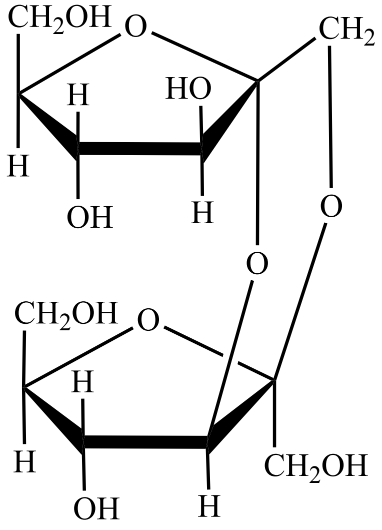

DFA-III

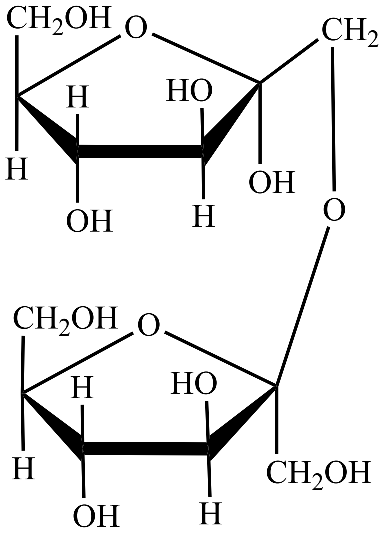

inulobiose

Fig. S1. The chemical structures of DFA-III and inulobiose.

```
   1  ATGAAATACCTGCTGCCGACCGCTGCTGCTGGTCTGCTGCTCCTCGCTGCCCAGCCGGCGATGGCCATGGCAGGAAAAAATTATTACGAT     90
      M  K  Y  L  L  P  T  A  A  A  G  L  L  L  L  A  A  Q  P  A  M  A  M  A  G  K  N  Y  Y  D

  91  GTAACAGAATGGAACGTGGGCGATCCGTATAAAGACATCGGCGAGGTGATCAACAGCATGCTGGCTGACATCAAAAGCAGGCAAACTGAC    180
      V  T  E  W  N  V  G  D  P  Y  K  D  I  G  E  V  I  N  S  M  L  A  D  I  K  S  R  Q  T  D

 181  AGTAATGTTGAGGAGGGTGGCAAGCCAGGTGCGGTTATCTACATTCCGAGCGGCGACTATCATCTGCGCACCCAGGTGGTTATAGACATC    270
      S  N  V  E  E  G  G  K  P  G  A  V  I  Y  I  P  S  G  D  Y  H  L  R  T  Q  V  V  I  D  I

 271  TCCTATCTAAAGATCATGGGTGCCGGTCACGGTTTTGTTTCTAGCTCGATTCGTTACAATCTGCCGAAGAGTGAGTGGGAAGATTTGCAC    360
      S  Y  L  K  I  M  G  A  G  H  G  F  V  S  S  S  I  R  Y  N  L  P  K  S  E  W  E  D  L  H

 361  GAGGTTTGGCCTGGCGGTAGCCGCATCCTGGTGGACCTTTCCCCGAAAGATGCGGACGGTGAAGCTGCGGGTGCGGCGTTTTACGTCGAG    450
      E  V  W  P  G  G  S  R  I  L  V  D  L  S  P  K  D  A  D  G  E  A  A  G  A  A  F  Y  V  E

 451  CGCTCCGGCAACCCGCGTATCTCCAGCGTGGAGTTCGAGAACTTCTGTATTGATGGTTTGCACTTCGCGGACGACGGCAGCGGTAAGAAC    540
      R  S  G  N  P  R  I  S  S  V  E  F  E  N  F  C  I  D  G  L  H  F  A  D  D  G  S  G  K  N

 541  GACCCAGAAAACACGTACGTTAACGGCAAGACCGGTATTTACGTCGCGTCTGCGCAAGATTCCTTTCGTATTACCGGCATGGGCTTCGTG    630
      D  P  E  N  T  Y  V  N  G  K  T  G  I  Y  V  A  S  A  Q  D  S  F  R  I  T  G  M  G  F  V

 631  TATCTGGAACATGGTGTTATCATCTACCACGCCGATGCCCTGAGCGTTCACGATAATTTCATCGCTGAATGTGGTAACTGCATCGAACTG    720
      Y  L  E  H  G  V  I  I  Y  H  A  D  A  L  S  V  H  D  N  F  I  A  E  C  G  N  C  I  E  L

 721  CGTGGTTGGGGTCAGGCAAGCAAAATCACTGACAACCTGATGGGTGCGGGCTATCGTGGTTACAGCGTATTCGCGCAGAATTTTGGTGGA    810
      R  G  W  G  Q  A  S  K  I  T  D  N  L  M  G  A  G  Y  R  G  Y  S  V  F  A  Q  N  F  G  G

 811  TTGCTGATTACGTCCAACAATATCTTTCCGAGAGGCACCAGCTGCGTGCACTTTGATAAAGTTGCGCGTTCCGTGATTACGGGCAATCGT    900
      L  L  I  T  S  N  N  I  F  P  R  G  T  S  C  V  H  F  D  K  V  A  R  S  V  I  T  G  N  R

 901  TTTCATAGCTTTTACCCGGGTGCGTTGGTTTTTGAGGGTAACTGCAGCGAGAACACCGTCAGCTCCAATCACTTCCTGCGTGATCATGAA    990
      F  H  S  F  Y  P  G  A  L  V  F  E  G  N  C  S  E  N  T  V  S  S  N  H  F  L  R  D  H  E

 991  CCGTGGGCACCGATGCTGAAAAACGATAACGGTCTCGACGATCGTTATGGCTTGTTACGCATCTCTGGCAATCACAATTCTGTCATTGCT   1080
      P  W  A  P  M  L  K  N  D  N  G  L  D  D  R  Y  G  L  L  R  I  S  G  N  H  N  S  V  I  A

1081  AATCATATTTCTGAAACCATTCATCGTCAGAGCATTAAGCCGGCTGGCGCGAAACCGGTGATCATTCATGTCGTGTCAGGCAAGGGCAAC   1170
      N  H  I  S  E  T  I  H  R  Q  S  I  K  P  A  G  A  K  P  V  I  I  H  V  V  S  G  K  G  N

1171  TATATCTCCGACAACCACATTGTGGCCACCACGGAAGCAGCGGATGCTCAAACCGGTGCCAGCGATAGTTGCTTCAGCGCTCAAGTGGAT   1260
      Y  I  S  D  N  H  I  V  A  T  T  E  A  A  D  A  Q  T  G  A  S  D  S  C  F  S  A  Q  V  D
                                                                        Lid region
1261  GCGCTGACCGCAACCGACAAGCTGGCAGTTCTGGACGTGACCACCGTTTTGGTAGCCAAAGAAAGCTTCCAGAACACGATTCTGGATTCT   1350
      A  L  T  A  T  D  K  L  A  V  L  D  V  T  T  V  L  V  A  K  E  S  F  Q  N  T  I  L  D  S

1351  GGTTCGGAAGAGCAGGTTGTGCTTGACCGCCGTGTTAATGCATTCCGCGCAACCCCGGTGCCGGGAGGCGTTCTCGAGCACCACCACCAC   1440
      G  S  E  E  Q  V  V  L  D  R  R  V  N  A  F  R  A  T  P  V  P  G  G  V  L  E  H  H  H  H

1441  CACCACTGA    1449
      H  H  *
```

Fig. S2. The nucleotide sequence and deduced amino acid sequence of *Bp*DFA-IIIase with a C-terminal His-tag. The predicted lid region is indicated by red color with underline.

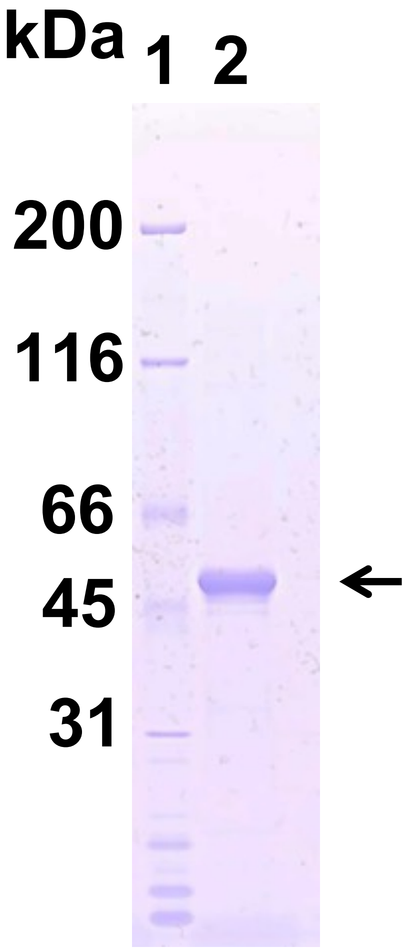

Fig. S3. SDS-PAGE of purified *Bp*DFA-IIIase. Lane 1, protein size maker and lane 2, *Bp*DFA-IIIase. Proteins and markers are stained with Coomassie Blue.

A

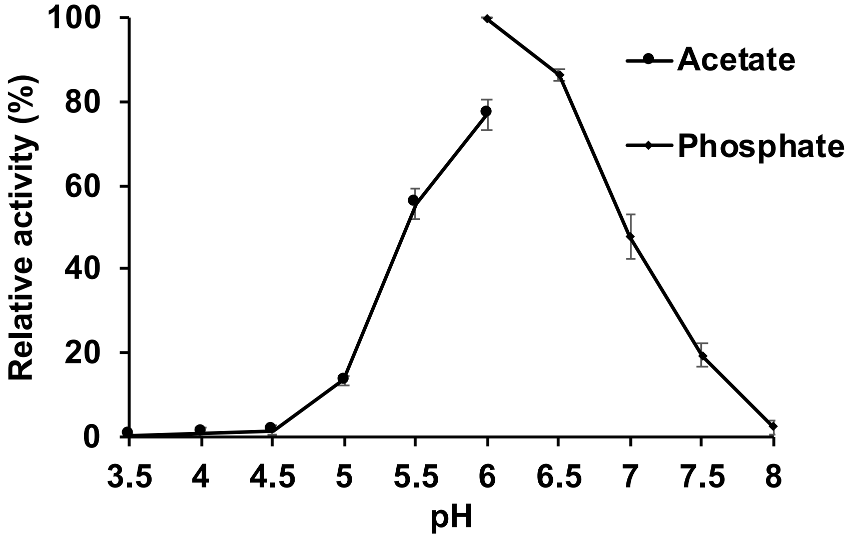

B

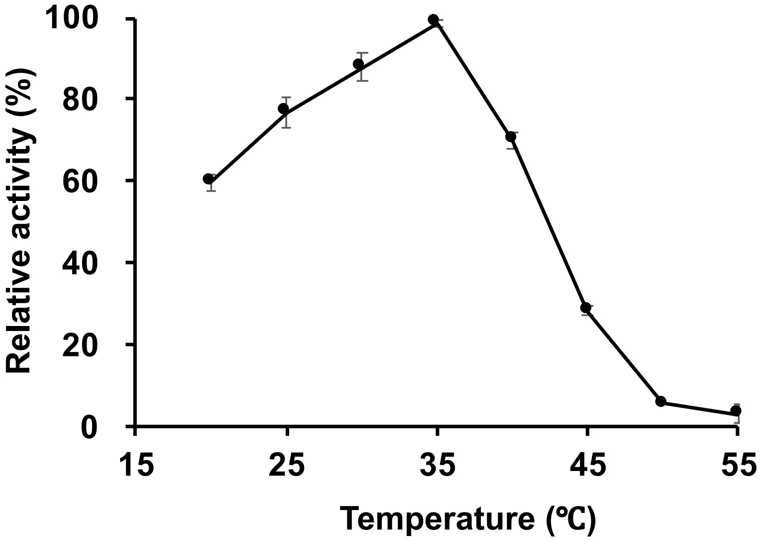

Fig. S4. Effect of pH and temperature on *Bp*DFA-IIIase activity. (**A**) Relative activity was evaluated at 37°C and different pH values. (**B**) Relative activity was evaluated at 20°C–55°C in sodium phosphate buffer (pH 6.0). Relative activity was expressed as a percentage of maximal enzyme activity. Values are means of three replicates ± standard deviation.
